# Ambiguous controls on simulated diazotrophs in the world oceans

**DOI:** 10.1038/s41598-022-22382-y

**Published:** 2022-10-22

**Authors:** U. Löptien, H. Dietze

**Affiliations:** 1 Department of Computer Science, Archaeoinformatics - Data Science, University of Kiel, Christian-Albrechts-Platz 4, 24118 Kiel, Germany; 2 MIN Faculty, CEN, Universität Hamburg, Grindelberg 5, 20144 Hamburg, Germany; 3 Department of Chemistry, King’s College London, 7 Trinity Street, London, UK

**Keywords:** Biochemistry, Biogeochemistry, Climate sciences, Ocean sciences

## Abstract

Nitrogen fixers, or diazotrophs, play a key role in the nitrogen and carbon cycle of the world oceans. Diazotrophs are capable of utilising atmospheric dinitrogen which is a competitive advantage over generally faster growing ordinary phytoplankton in nitrogen-depleted conditions in the sun-lit surface ocean. In this study we argue that additional competitive advantages must be at play in order to explain the dynamics and distribution of diazotrophs in the global oceans. Backed by growing published evidence we test the effects of preferential grazing (where zooplankton partly avoids diazotrophs) and high-affinity diazotrophic phosphorus uptake in an Earth System Model of intermediate complexity. Our results illustrate that these fundamentally different model assumptions result in a very similar match to observation-based estimates of nitrogen fixation while, at the same time, they imply very different trajectories into our warming future. The latter applies to biomass, fixation rates as well as to the ratio of the two. We conclude that a more comprehensive understanding of the competition between ordinary and diazotrophic phytoplankton will reduce uncertainties in model-based projections of the oceanic N cycle.

## Introduction

Nitrogen is an essential element in the metabolism of all organisms^1^. Even though it is much more abundant in air than for example carbon dioxide, the assimilation of carbon by phytoplankton in vast regions of the ocean is considered to be limited by the availability of nitrogen^2^. Among the reasons for this is that most phytoplankton cannot use the molecular nitrogen, that is so abundant in air and dissolved in sea water, because it cannot break the exceptionally strong chemical bond between the two nitrogen atoms, constituting molecular dinitrogen (N$_2$)^1^. An exception to this rule are so-called nitrogen fixing microorganisms (bacteria and archaea) that are capable of breaking this bond and, hence, can utilize molecular nitrogen (in addition to bioavailable nitrogen such as NO$_3^{-}$ and NH$_4^{+}$^3^). Their total input of bioavailable nitrogen to the ocean is substantial with current estimates ranging from 70 to 200 Tg N yr$^{-1}$^4–7^. Paleo-records suggest that for the last several 1000 years, this nitrogen input has been balanced by denitrification and anaerobic ammonium oxidation (anammox) which converts bioavailable forms of N under low-oxygen conditions back to N$_2$^8^. The apparent balance suggests a coupling mechanism between these sources and sinks of bioavailable N. Such coupling mechanisms on the overall oceanic N-budget are discussed controversially^7,9,10^—also because the controls on nitrogen fixation are, despite substantial research progress, not comprehensively understood. This, in turn, adds uncertainty to model-based projections into our warming future because the nitrogen cycle is intimately coupled to the production of greenhouse gases such as nitrous oxide and the biotic sequestration of carbon in the deep ocean^11^.

There is consensus that anthropogenic forcing, such as global warming and input of bioavailable nitrogen to the ocean, modulates the turnover of bioavailable nitrogen in the ocean^12^. This has been shown to trigger far-reaching consequences^13–16^. N$_2$-fixation adds extra nutrients to the system which ultimately maps onto an oxygen deficit at depth. While this in itself is of concern in already overfertilized coastal systems^17,18^ it can also trigger complex feedbacks where increasing oxygen minimum zones drive enhanced denitrification^19^. Further, it may be argued that this effect can be amplified by the fact that the N$_2$-fixing enzyme nitrogenase is deactivated by oxygen^20,21^ and that rising temperatures may promote both, more extended blooms and oceanic oxygen decline^22,23^.

The complexity of such feedbacks makes it challenging to predict the evolution of oceanic N-dynamics in our warming future. One way to set about here is to capture the respective dynamics in coupled ocean circulation models which explicitly resolve oceanic transport mechanisms (such as currents and mixing) along with the turnover of biogeochemical species (nutrients, carbon and oxygen)—with the aim to project the effects of changing environmental conditions and thereby facilitating management and mitigation measures. Such models rely on the identification of the key mechanisms and controls of the pelagic ecosystem. Typically these models do not resolve processes down to species level but, rather, represent average quantities of some functional groups in differential partial equations in order to reduce the complexity to feasible levels. This practical approach complicates a direct comparison to specific observations. Further, it introduces an element of subjectivity and uncertainty because there is no solid theoretical foundation on how to describe the net effects of a species community. Among associated challenges is the open question for the minimum complexity to be resolved^24^ and constraining so-called model parameters that govern the dynamics of the differential equations^25–27^. This puts this class of models apart from e.g. atmosphere and ocean models that are mainly built on first principles and are based on a much longer development than the relatively recent pelagic biogeochemical models^28^.

That said, a generic approach to capture the major aspects of the marine N-cycle in numerical models is to include an explicit representation of diazotrophs as extra functional group, that is distinctly different to the representation of ordinary non-fixing phytoplankton^27,29,30^. The underlying model assumptions and respective mathematical formulations vary from one model to another, but they typically agree on (1) diazotrophs are capable of utilising $\mathrm{N_2}$, while ordinary phytoplankton are not, and (2) diazotrophs grow slower (or at least not faster) than ordinary phytoplankton, because of the metabolic cost involved in providing means to break the strong bond between the two nitrogen atoms constituting $\mathrm{N_2}$^31^ . Further, diazotrophs apparently prefer higher temperatures^23^ and light levels^32^ for optimal growth (but the data on this is very diverse with different species featuring widely differing optima and new discoveries of diazotropic activities and habitats are continuously reported^18,33^). Finally, there is consensus that the availability of iron can limit $N_2$-fixation under iron-depleted conditions^34–36^.

A direct deduction of the generic assumptions is that diazotrophs only have an advantage over ordinary phytoplankton in regions where bioavailable nitrogen concentrations are below the limits of ordinary phytoplankton^37,38^, such as downstream of denitrification zones. In the remainder of the ocean, ordinary phytoplankton suppresses the relatively slow growing diazotrophs by outcompeting for resources, such as bioavailable phosphorus (P), iron and light^35,39–41^. Observations, however, indicate no such relation between nitrogen fixation and a nutrient supply that is low in nitrogen relative to, e.g., bioavailable phosphate^42,43^. Rather to the contrary, we find e.g. nitrogen fixation in the oligotrophic subtropical North Atlantic^44,45^ which is fuelled by high N:P ratios^46,47^. Further perplexity is added by the fact that diazotrophs are known to increase their internal N:P ratios via fixation beyond the needs of ordinary phytoplankton. Thereby they ultimately overstock the system with extra nitrogen (altering the near-surface N:P ratios)—which downsizes the extent of their very own ecological niche^19^. Following this line of thought foretells that, in these models, diazotroph abundance is limited to regions which are low in N and have excess P (such as downstream from denitrification/suboxic regions) and, further, that diazotrophs shrink their very own habitat by reducing excess P (even without explicitly formulating a nitrate-replete-handicap)—unless diazotrophs are given an additional competitive advantage that can compensate for their typically rather slow growth rates.

Here we discuss two such potential advantages suggested in the literature: (1) selective grazing by zooplankton, where ordinary phytoplankton is exposed to higher grazing pressure than the (slower-growing) diazotrophs^30,48–50^ and (2) higher competitiveness for inorganic P, as suggested by some observations^51,52^, and as implemented, e.g., in the Baltic Sea model SCOBI which assumes a lower half-saturation constant for P for nitrogen fixing cyanobacteria than for diatoms^53^ and in the BALTSEM-model, which uses a lower half-saturation constant for cyanobacteria than for summer species^54^. Note that the latter, seemingly small, parameter adjustment entails major implications: fixing nitrogen above their needs ultimately swamps the system with N which can be used by their competitors and thereby finally rendering P more limiting than N even for ordinary phytoplankton. (An example of this has been documented in the oligotrophic subtropical North Atlantic where near-surface nitrogen fixation accumulates as N excess in the thermocline^44,45^ such that the N:P ratio of the upwards diffusive nutrient fluxes from the thermocline to the nutrient consuming surface layer are higher than the requirements of other phytoplankton^46^). By reducing the half-saturation constant for P, diazotrophs become more competitive in a self-inflicted environment. Once dominant, diazotrophs could then use subsequent nutrient (P)-pulses to their advantage.

In this study we explore the above described parameter uncertainties and, thus, map out a lower bound of the uncertainty of projected diazotrophic biomass and fixation that is associated with our incomprehensive understanding of respective controls^55^. To this end we focus on selective grazing and higher competitiveness for inorganic P, while other mechanisms and uncertainties such as temperature effects and controls of iron limitation^34^ are not considered here. Among the reasons are difficulties in representing the wide variety of diazotrophs and the iron cycle in models. E.g. recent studies^56–59^ report surprising evidence for nitrogen fixers in colder, polar regions of both hemispheres while it was previously assumed that diazotrophs favour higher temperatures (in our model a temperature limit for the occurrence of diazotrophs limit is set to 15$^{\circ }$). As for the iron dynamics there is evidence that a lack of iron can limit the growth of diazotrophs^35^ and altering the iron input to the ocean may trigger effective feedbacks^60^. The inclusion of related processes in contemporary models, however, remains difficult. The present-day observational base is so sparse that the current recommendation^61^ for CMIP6-models is to initialize the models with “.... the median model result from the Iron Model Intercomparison Project” FeMIP^62^ rather than using sparse observational products.

Our analyses are based on an Earth System model of intermediate complexity which includes diazotrophs as extra functional group^50^. The reference model is based on the generic assumptions that diazotrophs do not rely on the presence of nitrogen in their surrounding waters and grow comparably slow, especially at low temperatures. These basic pillars of the model are refined by adding selective grazing and a higher competitiveness for inorganic P to individual model versions. Our two respective model versions are dubbed GRAZ and OLIGO. Both the grazing and the competitiveness are determined by adjusting model parameters which we choose such that an optimal fit to the pre-industrial nitrogen fixation estimate^7^ for quasi-equilibrated model states under pre-industrial $CO_2$ emissions is reached (details on the implementation and parameter settings are provided in the supplement). The respective adjustments refer to the half saturation constant of phosphate uptake ($k_P^d$), the food preference of zooplankton ($\theta _d$) and the growth handicap of diazotrophs ($C_d$) (cf. Tab S1 in the supplement). In addition we run a control simulation (CONTR) where we switch off the selective grazing (that is inherent already in the reference model^50^) and where the competitiveness for inorganic P is identical for diazotrophs and ordinary phytoplankton, such that the ability of diazotrophs to fix atmospheric nitrogen is their only competitive advantage. The growth handicap of diazotrophs was adjusted in CONTR to match GRAZ and OLIGO. Further, we present results from a model experiment, named DECAY which showcases that in the absence of denitrification diazotrophs would die out without further competitive advantages than their ability to fix nitrogen, because they gradually fill up nitrogen deficits globally. (Details on the implementation and parameter settings are provided in the supplement). Projections with GRAZ and OLIGO covering the period 1800-2150 along an RCP 8.5^63^ emission trajectory illustrate the implications of one paradigm versus the other.

## Results

### Historical climate

**Figure 1:**
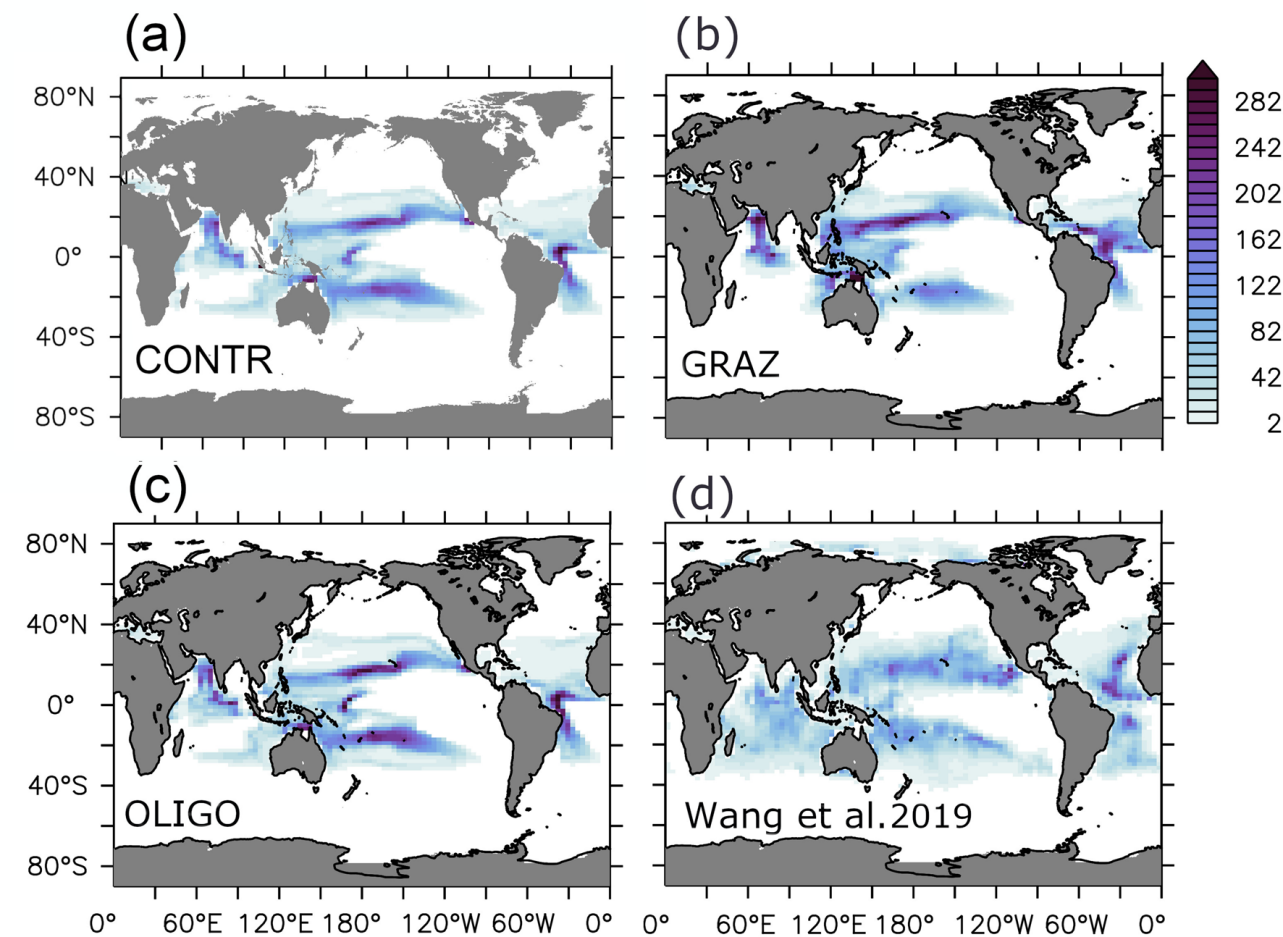
Annual mean nitrogen fixation rate in the water column in units mmol N/m$^2$/yr. (**a**–**c**) refer to simulations CONTR, GRAZ and OLIGO, respectively. (**d**) refers to an observation-based estimate^7^.

**Figure 2:**
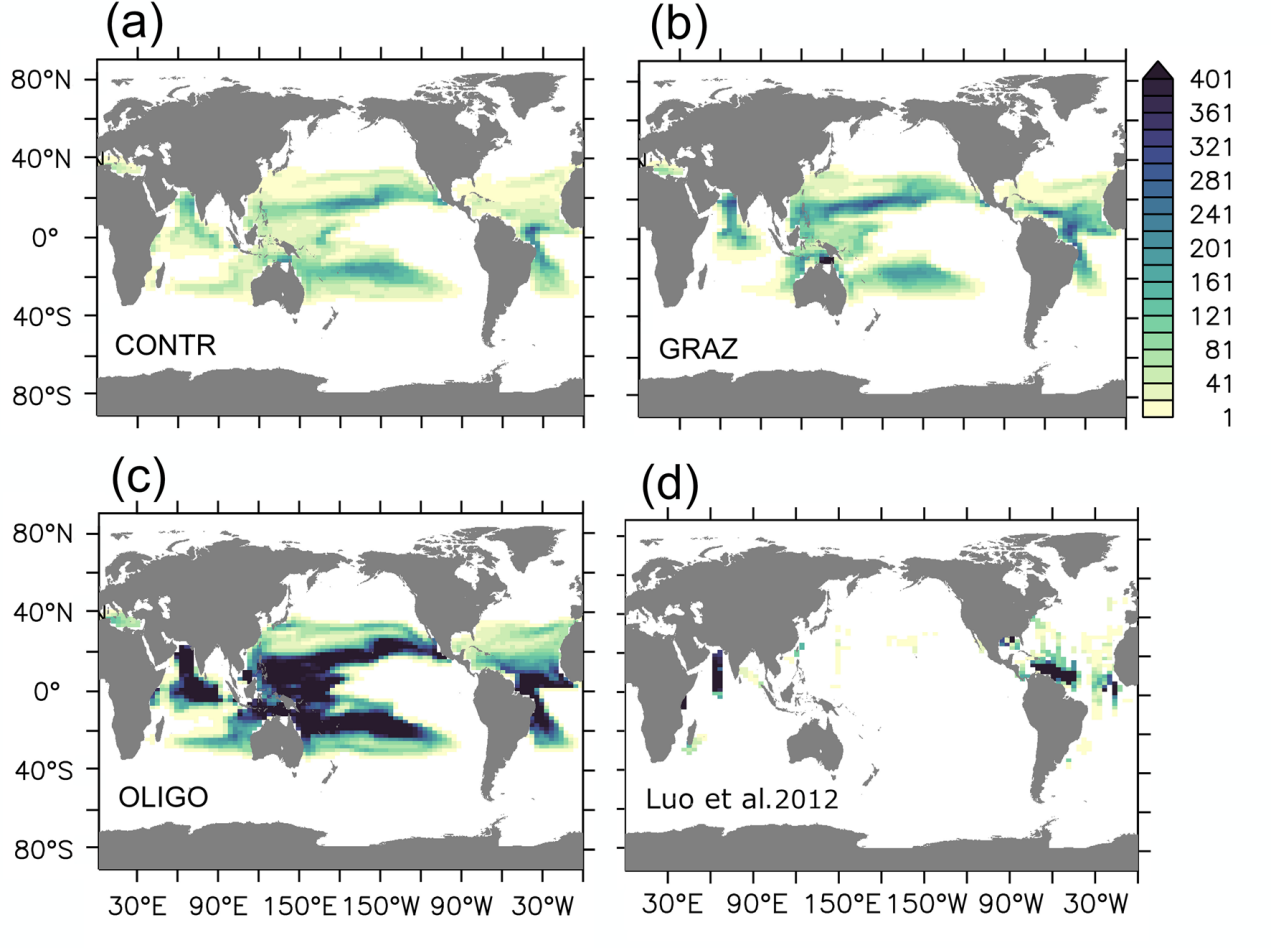
Annual mean diazotrophic biomass inventories in the upper 100 m in units mg C/m$^2$: (**a**–**c**) refer to simulations CONTR, OLIGO and GRAZ, respectively. (**d**) is based on observations^67^, interpolated linearly over depth and then gridded onto the model grid.

Our control simulation CONTR does not assign any competitive advantage to diazotrophs other than that they can grow in in the absence of nitrate while the competing ordinary phytoplankton can not. Consequently, simulated nitrogen fixation and diazotroph abundance is restricted to those areas where water with a history of denitrification reaches the sun-lit surface. In our model setup (as in many others^19,64,65^) these regions are the tropical Atlantic (which, however, is spurious^65^), tropical Pacific and the Indian Ocean (Figs. 1a and 2a). Consistent with this result we find that if we shut off all denitrification in the CONTR configuration (by deleting the respective term in the partial differential equation; experiment DECAY) diazotroph biomass decreases exponentially (as illustrated in the supplement in Fig. S1). The timescale of the decline is set by the ratio between the global P inventory in excess to its Redfield equivalent of nitrogen and the nitrogen fixation rate which reduces that P excess inventory: as the fixation fills in the nitrogen deficit the niche for diazotrophs closes in (because all other competitive advantages of diazotrophs are shut off in CONTR). Based on global fixation estimates ranging from 70 to 200 Tg N yr$^{-1}$^4–7^ and a P excess equivalent of 5.5 P mol N^66^, the timescale of the disappearance of the niche for diazotrophic is 400–1100 years—with the latter estimate matching our simulated exponential decay in experiment DECAY.

Figures 1 and 2 show simulated fixation rates and diazotrophic biomass, respectively. CONTR, GRAZ and OLIGO all show very similar patterns of fixation. In terms of a quantitative misfit metric describing the difference to a recent data-based reanalysis of fixation rates^7^ we find a root mean squared error of 36, 36 and 35 mmol N/m$^2$/yr for CONTR, GRAZ and OLIGO, respectively (and allimproved compared with the 55 mmol N/m$^2$/yr of the original UVic 2.9 reference version^50^). A comparison with observed diazotrophic biomass^67^ suggests that OLIGO is more realistic than GRAZ which apparently features an unrealistically low biomass: For example, taking the upper 10 percentiles as a measure for bloom intensities (derived from the respective histograms of positive local annual mean values), OLIGO lies with 716 mg C/m$^2$ much closer to the observations (598 mg C/m$^2$ ) than GRAZ (282 mg C/m$^2$) and the original UVic 2.9 (320 mg C/m$^2$). Respective histograms are depicted in the supplement (Fig. S2). In this context we have to stress that stronger selective grazing on other phytoplankton has the potential to increase the biomass of diazotrophs substantially (within the explored parameter range up to almost one order of magnitude compared to the original UVic 2.9). This enhancement, however, worsens in our model the fit to the observation-based estimate for nitrogen fixation). CONTR is even more unrealistic in that it features a global biomass which is reduced by a factor of two relative to GRAZ. Further we find that diazotrophs would die out in CONTR in the absence of denitrification (see experiment DECAY described in the suplememt) which is—as we argue in the introduction—inconsistent with observations of high diazotrophic abundances in the subtropical North Atlantic.

The caveat being here, however, that the observational database is sparse. Further, the simulated biomass is rather climatological while the observations are anecdotal taken from an environment that is driven by large fluctuations on small spatial and temporal scales.

Further investigation into, e.g., simulated surface nutrients give a rather inconclusive picture and the differences between the model versions lead to relatively small values when compared to overall uncertainty and misfits^68^: Based on the World Ocean Atlas 2005^66^ point values we calculate a root mean squared errors of 2.5 mmol NO$_3$/m$^3$ and 2.6 mmol NO$_3$/m$^3$ for OLIGO and GRAZ respectively. For surface phosphate we calculate 0.29 mmol PO$_4$/m$^3$ and 0.26 mmol PO$_4$/m$^3$ for OLIGO and GRAZ, respectively. A visual impression of the simulated surface nutrients in the different model versions is provided in the supplement (Fig. S3).

**Figure 3:**
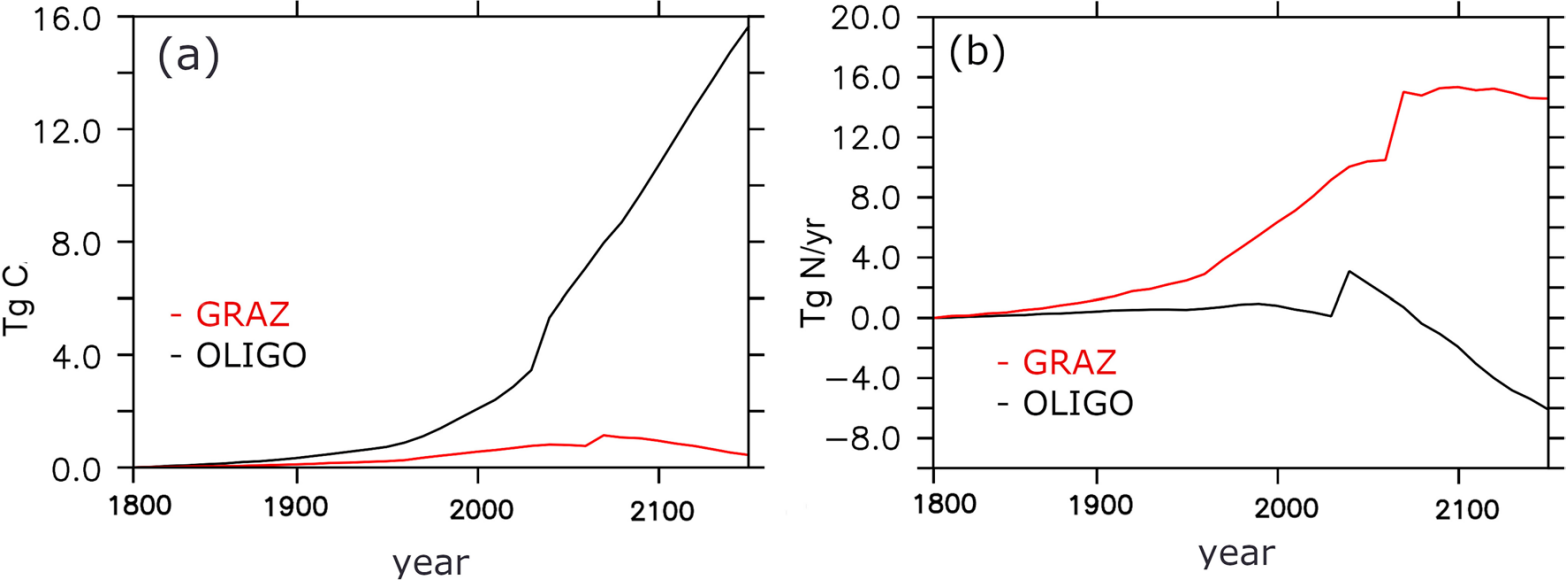
Projected evolutions along an RCP 8.5^63^ emission trajectory referenced to 1800 as simulated with configurations GRAZ (red lines) and OLIGO (black lines). (**a**) refers to global annual mean diazotrophic biomass in units Tg C. (**b**) refers to global annual mean nitrogen fixation in units Tg N yr$^{-1}$.

The largest differences identified between the contemporary climatologies simulated with OLIGO and GRAZ are in the ratio between diazotrophic biomass and global fixation rate: OLIGO features a factor of two higher biomass to fixation ratio than GRAZ. One reason is that in OLIGO diazotrophs can persist even under extremely nutrient-depleted conditions because they can cope with lower P concentrations than ordinary phytoplankton. Likewise, diazotrophs can take over from ordinary phytoplankton once phosphate concentrations are depleted e.g. at the end of a spring bloom. We speculate that this effect is amplified by the fact that the effective grazing pressure is much lower under extremely oligotrophic conditions. However, even though the diazotrophs in OLIGO may occupy larger areas or more of the seasonal cycle this does not necessarily provide an opportunity to score high fixation rates because the ultra-oligotrophic conditions do provide only for little net growth.

Another difference between OLIGO and GRAZ surfaced when regarding the robustness of the underlying model formulation, expressed in terms of respective parameter sensitivities. Specifically, we compare the sensitivity of changes in the half saturation constant of phosphate uptake ($k_P^d$) in configuration OLIGO versus changes in the food preference ($\theta _d$) in configuration GRAZ. Changing $\theta _d$ and $k_P^d$, by 20% of their covered range (0.02 and 0.0088, respectively) yields, on average, a much larger change in the amount of fixed nitrogen for the *selective grazing* paradigm, compared to *low P-limit* paradigm (16.8 and 0.8 Tg N yr$^{-1}$, respectively). For the integrated biomass of diazotrophs, however, the respective changes are similar for both paradigms (4.9 Tg C for *selective grazing* v.s. 5.8 Tg C for *low P-limit*). Note, in this context that for both paradigms, the biomass of diazotrophs yields a highly nonlinear response to parametric changes. As a side aspect we found, inline with previous results with another model^7^, that decreasing the food preference for diazotrophs can even lead to a tipping point whereafter all diazotrophs are constrained to upwelling regions. We conclude that the paradigm *low P-limit* produces a model behavior that is much more robust towards parameter changes than the *selective grazing* paradigm, in the sense that small changes in (rather unconstrained) model parameters effect rather moderate changes in the quality of the fit to observations. In contrast, small changes to the *selective grazing* paradigm effect in large changes or may even induce a regime shift. Overall, however, we found the performance of OLIGO and GRAZ to be surprisingly similar—given that the underlying paradigms that define the respective niches of diazotrophs are so different.

### Future projections

In simulations of an RCP 8.5 emission scenario we investigated if the similarity of OLIGO and GRAZ extends to projected trajectories into our warming future. This gives guidance on the question to what extent contemporary model fidelity is indicative of that uncertainty that is associated with the ambiguity of controls on diazotrophs. We find that the RCP 8.5 projections of both, OLIGO and GRAZ, are closely tied to an increasing vertical stratification, effected by an ocean that is warmed from above. The water expands at the surface and this increases the vertical density gradient, such that the energy requirements for vertical mixing are increased (because mixing is now associated with pushing lighter, more buoyant water downwards). The increased energy demands for mixing result in a dampening of mixing events and, overall, in less nutrients mixed upwards to the sun-lit surface. Among the processes setting in are (1) a reduction of phosphate (and nitrate) supply to the surface (globally and in oligotrophic regions), (2) an increase of oligotrophic regions where phosphate concentrations are depleted at the surface. (Note that the areas affected by iron limitation remain unchanged in our model. Hence, given the unknowns in iron dynamics and iron limitation of diazotrophs, we conclude that our approach provides a lower bound of uncertainty).

The increase in the size of the oligotrophic regions is captured well in both model versions: we define oligotrophic regions as regions featuring a chlorophyll *a* concentration of less than 0.07 mg Chl a m$^{-3}$ at the surface^69^. Based on this threshold and applying a constant conversion factor of 1.59 mg Chl a mmol N$^{-1}$^70,71^ we find that oligotrophic regions expand by 92% and 90% from 2000 to 2100 in OLIGO and GRAZ, respectively—both of which are in the same order of magnitude as the 1998 to 2006 satellite based estimate of^69^ (19% per decade). Differences arise, however, because the underlying paradigms of OLIGO and GRAZ yield very different responses to these changes—even though they share a very similar behavior under contemporary conditions: in the simulation OLIGO, the diazotrophs can take advantage of the increasing vertical stratification because the diazotrophs are expertly exploiting oligotrophic conditions. The black line in Fig. 3a shows that the biomass of diazotrophs increases globally along with an increase in size of the oligotrophic regions. In addition, Fig. 3b illustrates that, even though the size of oligotrophic regions and the diazotrophic biomass increases, the nitrogen fixation decreases in OLIGO. The reason is a reduced total supply of phosphorous to the surface in (expanded) oligotrophic regions which, in our model, puts an upper limit on nitrogen fixation.

In contrast, the development of nitrogen fixation and abundance of diazotrophs goes much more hand-in-hand in GRAZ, and the projected evolution of both variables differs strongly from OLIGO: an initial increase in both variables is followed by a subsequent decay. Such switching behaviour at rather arbitrary tipping points (that are set by the model parameters) are typical for the generic zooplankton formulation used here and have been described earlier^25,72^ (their “Future projections” section).

More specifically we find, referenced to year 2000, that the difference between the respective globally averaged trends of the biomass of diazotrophs in OLIGO and GRAZ becomes significant by 2050. An exploration of local changes (Fig. S4) reveals a close coupling to changes in denitrification rates which increase in response to warming. For example, both OLIGO and GRAZ show increased fixation rates and diazotrophic biomass in the Bay of Bengal where both configurations feature enhanced denitrification rates. Similarly, both OLIGO and GRAZ show increased fixation and diazotrophic biomass in a rim around the equatorial Pacific. Similarly, because the rim is fuelled by equatorial upwelling which taps into N-depleted waters originating from a denitrifying oxygen minimum zone that is expanding in our model along with global warming.

Apart from the similarities driven by similar responses to denitrification differences are apparent in Fig. S2 and can be attributed back to the conceptual differences in OLIGO and GRAZ. For example, OLIGO features a stronger increase of diazotrophic biomass than GRAZ in the Indic and Pacific even though respective denitrification rates increase less in OLIGO than in GRAZ. Further and more prominently in the oligotrophic subtropical North Atlantic the diazotrophic biomass increases in OLIGO while the respective average decreases in GRAZ.

We summarize that GRAZ and OLIGO yield very similar fidelities that exceed those of previous standard model configurations^50^ when compared with contemporary estimates of nitrogen fixation. Given the complex highly non-linear coupled entanglement of biogeochemical processes and ocean circulation we find, however, that on trajectories into our warming future the similarity between GRAZ and OLIGO breaks. We conclude that a reconciliation of to-date ambiguous controls on diazotrophy must precede reliable projections of the nitrogen inventory.

## Discussion

We investigate the impact of model parameter changes on simulated diazotrophs and nitrogen fixation. The focus is on changes relative to the parameters of ordinary phytoplankton, which might extend the ecological niche of diazotrophs beyond nitrogen-depleted regions. We explore both, the fidelity to reproduce contemporary data and on projections into our warming future. Our results are based on a numerical Earth System Model of intermediate complexity.

We find that introducing very different mechanisms (or paradigms) that have previously been suggested in the literature^55^ fit a recent and comprehensive observation-based estimate of nitrogen fixation^7^ equally well. Given that, in addition, there are significant differences between such observation-based estimates^7,18,37^ we conclude that these current data are insufficient to dissect the ambiguous controls on diazotrophy in the global ocean. Further, we found model-data misfit metrics based exclusively on fixation rates to be rather uninformative as concerns the representation of diazotrophs in the model, because fixation rates and the abundance of diazotrophs can be decoupled. This is unfortunate because comprehensive observational estiamtes do currently exist only for rates^7,18,37^ while products of diazotrophic biomass—despite substantial community accomplishments ^67^—are still too sparse to reliably rank one process over another. Further perplexity is added by a recent study which suggests that the diazotroph *Trichodesmium* can be polyploidy which additionally puts a question mark on some of the observational data that is based on nifH gene analyses^73^.

Depending on the two paradigms presupposed here, we find two very different trajectories into our warming future (considering a RCP 8.5 emission scenario): Presupposing that preferential grazing is the major competitive advantage (besides the ability to utilize $N_2$) extrapolates to a modest increase in diazotrophic biomass associated with a large increase ( > 10 Tg N yr$^{-1}$) in global fixation rate. Essentially, the reduced grazing pressure allows the diazotrophs to populate top-down controlled regions with relatively ample nutrient supply. This boosts diazotrophic productivity and increases the ratio between changes in fixation rate and changes in diazotrophic biomass.

On the other hand, presupposing that an enhanced competitiveness under oligotrophic (P-limited) conditions is the major competitive advantage (besides the ability to utilize $N_2$) extrapolates to a large increase in diazotrophic biomass and, surprisingly, to an eventual decline of nitrogen fixation rate. The increase in biomass is driven by the increase in oligotrophic regions where the warming-induced increase in stratification inhibits nutrient supply from the nutrient replete abyss to the nutrient-depleted sun-lit surface ocean. However, even though diazotrophs increase their habitat their productivity decreases because of the net total decreasing nutrient supply to (expanding) oligotrophic areas. In our simulation this effect is so strong that the ratio between changes in fixation rate and changes in diazotrophic biomass switches sign as the earth warms.

In summary we find for very different paradigms: (1) an equal fidelity of simulation when compared with contemporary observational estimates, (2) a highly diverse increase in projected diazotrophic biomass, and (3) changes of opposing sign for projected global nitrogen fixation rates. Combining both paradigms leads to model solutions that cluster around the respective reference simulations depending on the relative effect of respective model parameter settings over one another (as shown in the supplement Fig. S5). This showcases the need for a more comprehensive understanding of the competition between ordinary phytoplnakton and diazotrops—if uncertainties of anticipated changes in the global oceanic N-cycle are to be constrained. Specifically, we find that the contemporary observational estimates are either too sparse or inconsistent^7,37^ to constrain the formulations of the dynamics of diazotrophs and ordinary phytoplankton in Earth System models such that their projections of fixation become robust.

Caveats apply and, hence, the scale of uncertainty showcased here must be understood as a lower bound. The model we use is of intermediate complexity and neglects potentially important and highly non-linear processes. Among those is atmospheric variability, small-scale processes, effects of iron dynamics and non-Redfield stoichiometric processes.

## Methods

The sensitivity experiments were conducted with the UVic 2.9 Earth System Model of intermediate complexity^38,50^. For this study we use four different model versions, advancing an initial approach outlined in the respective preprint^74^:

In all model versions phytoplankton growth is controlled by the availability of light and nutrients (here, nitrate, phosphate and iron, where the effect of the latter is parameterized by prescribing a mask, rather than explicitly resolving respective dynamics prognostically). The actual growth rate of non-diazotrophic phytoplankton, $J_{O}$, is, in case of low irradiance (I) and/or nutrient-depleted conditions, the maximum potential rate $J_o^{max}$ reduced by the following implementation of Liebig’s law of the minimum:

1 $$\begin{aligned} J_{O}=min\left( J_{IO}, J_O^{max} \frac{NO_3}{k_N+NO_3}, J_O^{max} \frac{PO_4}{k_P+PO_4}\right) . \end{aligned}$$

where $k_N$ and $k_P$ are the *half saturation constants* for $NO_3$ and $PO_4$, respectively.

The actual growth rate of diazotrophs $J_D$ is similar but differs in that it is not affected by nitrate deficiency:

2 $$\begin{aligned} J_{D}=min \left( J_{IO}, J_D^{max} \frac{PO_4}{k_P^d+PO_4} \right) . \end{aligned}$$

 Phytoplankton blooms are typically terminated by zooplankton grazing once the essential nutrients are depleted such that phytoplankton growth does not longer keep up with the grazing pressure, that has built up during the bloom. The reference version of our Uvic 2.9 is an implementation of the preferential grazing paradigm^50^ where grazing is defined by a multiple-prey Holling II functional response that assigns preferences for different types of prey (phytoplankton, detritus and zooplankton). The rate of grazing on phytoplankton is determined by:

3 $$\begin{aligned} Graze = \mu _{max} \cdot Z \cdot \theta \cdot P, \end{aligned}$$

with a maximum growth rate $\mu _{max}$ = 0.4 d$^{-1}$ and *Z* referring to the biomass of zooplankton. Grazing on ordinary phytoplankton, $P_O$, is calculated as in the original UVic 2.9 model by setting $\theta =\theta _o=0.3$. Grazing on diazotrophs, $P_D$, is calculated with a lower grazing preference $\theta =\theta _d=0.1$.

Our (only slightly) modified model version OLIGO uses a lower half saturation constant of phosphate uptake for diazotrophs than the standard UVic 2.9 version^50^. The model version GRAZ, on the other hand, uses the original half saturation constant of phosphate uptake (identical for diazotrophs and ordinary phytoplankton) and assigns a lower food preference of zooplankton for diazotrophs than for ordinary phytoplankton—a paradigm which is already implemented in the standard UVic 2.9 model^50^ while the GRAZ model version differs in terms of the strength of this effect: in the GRAZ-model the respective model parameter has been adjusted to match the recent nitrogen fixation estimate^7^ as close as possible (such that they excel the performance of the standard UVic 2.9 model^50^). This approach has been chosen to make the model results of GRAZ and OLIGO comparable. In addition to the two optimized model setups described above, we performed two control simulations: (1) The setup CONTR assumes the same grazing pressure on diazotrophs and other phytoplankton and also uses the same half-saturation constants for dizotrophs and ordinary phytoplankton. (2) The setup DECAY is identical to CONTR with the only difference being that denitrification is artificially set to zero by nulling the respective term in the equations.

All model versions were integrated to quasi-equilibrium under pre-industrial emissions. To compare their respective sensitivities the model versions GRAZ and OLIGO are projected into the future (along a RCP 8.5 36 emission trajectory^63^). Further details about the model and the different setups and a table with all parameter settings are provided in the supplement.

## Supplementary Information

Supplementary Information.

## Data Availability

The model output is archived at 10.5281/zenodo.7234180. The data are distributed under the Creative Commons Attribution 4.0 License. The observational data collected by Luo et al. (2012) are available via https://doi.pangaea.de/10.1594/PANGAEA.774851.
