# Supplementary material for: Ambiguous controls on simulated diazotrophs in the world oceans
Source: Sci Rep. 2022 Oct 22;12:17784. doi: 10.1038/s41598-022-22382-y (PMC9588038; doi:10.1038/s41598-022-22382-y)
Supplement: Supplementary file 1 — Supplementary Information. [file 41598_2022_22382_MOESM1_ESM.pdf]

Ambiguous controls on simulated diazotrophs in the world oceans

U. Löptien[1,2], H. Dietze[1,3]

[1] *Department of Computer Science, Archaeoinformatics - Data Science, University of Kiel, Christian-Albrechts-Platz 4, 24118, Kiel, Germany*

[2] *MIN Faculty, CEN, Universität Hamburg, Grindelberg 5, 20144 Hamburg, Germany*

[3] *Department of Chemistry, King's College London, 7 Trinity Street, London, UK*

Observational Data I*n situ* observations of diazotrophic biomass and nitrogen fixation rates [1], are very useful but still too sparse to allow for robust comparisons between models and observations. This has been a serious drawback hindering the identification of the major controls of diazotrophy. This study exploits arguably the most recent and most comprehensive product [2], that fills data gaps by combining observations with inverse biogeochemical and prognostic ocean modeling - an approach used successfully earlier [3].

The Reference Model We use the UVic 2.9 Earth System Model [4,5]. UVic 2.9 is of intermediate complexity, relatively low computational cost and thus allows us to perform a multitude of quasi-equilibrium simulations which is essential for our approach. UVic 2.9 has been used in many studies [6–11], including those studying diazotrophy [12,13]. Note, that different flavours of UVic 2.9 exist and we refer here to the extensively documented reference version [4]. The horizontal resolution is 1.8° in latitude and 3.6° in longitude in all submodules. It includes a single-level atmospheric energy-moisture balance model (with prescribed surface winds from the NCAR/NCEP monthly climatology), a dynamic-thermodynamic sea ice model, a simple land ice model and a prognostoc terrestrial vegetation component [14].

The ocean component of UVic 2.9 is based on a three-dimensional primitive-equation model [15]. The vertical discretization of the ocean comprises 19 levels and increases gradually from $50\,m$ at the surface to $500\,m$ at depth. The vertical background mixing parameter, κ_h, is 0.15 cm^2s^{-1} throughout the water column. In the Southern Ocean (south of 40°S) this background diffusivity, κ_h, is increased by 1.0 cm^2s^{-1}. An anisotropic viscosity scheme [16] is implemented to improve the equatorial circulation [17]. A marine pelagic biogeochemical model is coupled to the ocean circulation component. Its prognostic variables are ordinary (i.e. non-diazotrophic) phytoplankton (P_o), diazotrophic phytoplankton (P_d), zooplankton (Z), detritus (D), nitrate (NO_3), phosphate (PO_4), dissolved oxygen (O_2), dissolved inorganic carbon (DIC) and alkalinity (ALK). Note that the dispersive numerics of the oceanic advection occasionally produces small negative values in phytoplankton and zooplankton concentrations. These values are set to zero in the model evaluation.

In the following, we present a choice of details relevant for the simulated dynamics of diazotrophs (relevant model parameters are listed in Tab.S1 while relevant model data misfits are listed in Tab. S2): phytoplankton growth is generally controlled by the availability of light and nutrients (here, nitrate, phosphate and iron, where the latter is parameterized by a prescribed mask, rather than explicitly resolved). Phytoplankton blooms are terminated by zooplankton grazing, once the essential nutrients are depleted and the related reduction in phytoplankton growth does not longer keep up with the grazing pressure, that has built up during the bloom. For ordinary phytoplankton the maximum potential growth rate is

$$J_o^{max} = a \cdot (\frac{Fe}{k_{Fe} + Fe}) exp(T/15.65), \tag{1}$$

where k_{Fe}=0.1 $mmol\ Fe/m^3$ is the *half-saturation constant* for iron (Fe)-limitation and $a = 0.6\,d^{-1}$ determines the maximum potential growth rate at an ambient temperature $T = 0°C$.

For diazotrophs the formulation of the maximum potential growth is similar, but diazotrophs are permanently disadvantaged in nitrate-replete waters by $C_d = 0.3$. In addition, growth is stalled

in waters colder than $15°C$:

$$J_D^{max} = C_d \cdot a \cdot max\left(0, \frac{Fe}{k_{Fe}+Fe} \cdot exp(T/15.65) - 2.6\right). \tag{2}$$

The actual growth rate of non-diazotrophic phytoplankton, J_O, is, in case of low irradiance (I) and/or nutrient depleted conditions, the maximum potential rate J_o^{max} reduced by the following implementation of Liebig's law of the minimum:

$$J_O = min\left(J_{IO}, J_O^{max}\frac{NO_3}{k_N+NO_3}, J_O^{max}\frac{PO_4}{k_P+PO_4}\right), \tag{3}$$

where k_N and k_P are the *half saturation constants* for NO_3 and PO_4, respectively.

The actual growth rate of diazotrophs, J_D, is similar but it is not affected by nitrate deficiency:

$$J_D = min\left(J_{ID}, J_D^{max}\frac{PO_4}{k_P^d+PO_4}\right), \tag{4}$$

The light-limited growth J_{IO} and care both determined by:

$$J_{IOandD} = \frac{\alpha I J_{OandD}^{max}}{(\alpha I)^2 + (J_{OandD}^{max})^2}. \tag{5}$$

The initial slope of the P-I curve (α) is set to 0.1 $(W/m^2)^{-1}d^{-1}$. Diazotrophs take up available nitrogen following:

$$NO3_{upt} = min\left(J_{ID}, \frac{min(NO_3, PO_4 \cdot 16)}{(k_P^d \cdot 16) + min(NO_3, PO_4 \cdot 16)} \cdot J_D^{max}\right) \cdot P_D, \tag{6}$$

where P_D denotes the biomass of diazotrophs (in units $molN/m^3$). When no or not enough bioavailable N is available, nitrogen fixation tops the respective N pool up to a Redfield-ratio of N:P=16. Note that the above formulation differs slightly from the original formulation [4] which reads:

$$NO3_{upt} = min\left(J_{ID}, \frac{PO_4}{k_P^d + PO_4} \cdot J_D^{max}\right) \cdot \frac{NO_3}{(k_P^d \cdot 16) + NO_3} \cdot P_D, \tag{7}$$

Our minor change ensures a more realistic behavior where no nitrogen is fixed under nitrate-replete conditions. When applied to the reference model version, the difference to the original simulation turned out to be negligible.

The reference version of our Uvic 2.9 is an implementation of the preferential grazing paradigm [4]. The grazing is characterized by a multiple-prey Holling II functional response that assigns preferences for different types of prey (phytoplankton, detritus and zooplankton). The rate of grazing on phytoplankton is determined by:

$$Graze = \mu_{max} \cdot Z \cdot \theta \cdot P, \tag{8}$$

with a maximum growth rate μ_{max} = 0.4 d^{-1} and Z referring to the biomass of zooplankton. Grazing on ordinary phytoplankton, P_O, is calculated by setting $\theta = \theta_o = 0.3$. Grazing on diazotrophs, P_D, is calculated with a lower grazing preference $\theta = \theta_d = 0.1$.

Model setups and specific assumptions

The implementations of the two competitive advantages on the ecological niche of diazotrophs investigated in our study refer to specific parameter settings in our model. The *selective grazing* paradigm is implemented by adjusting the grazing preferences for diazotrophs and ordinary phytoplankton, θ_d and θ_o, with the additional constraint $\theta_d + \theta_o = 0.4$. In addition, C_d is varied in attempt to minimize the misfit to observations. In total, we explored 24 combinations of the parameters θ_d, θ_o and C_d. In the following, GRAZ refers to the most realistic member of the set of 24 that outperforms the reference version (cf., Tab. S1 & S2). Note that also the original simulation [4] uses *selective grazing* but is not optimized towards a nitrogen fixation estimate (which is necessary for our purposes, i.e., the comparison with the second mechanism). Preferential grazing is a relatively common assumption in biogeochemical models and e.g. related to the fact that some diazotroph species can be inedible or toxic to zooplankton [18,19].

The second paradigm, represented by OLIGO, assumes that diazotrophs can cope better with oligotrophic conditions than ordinary phytoplankton. This paradigm is based on the the idea put forward by foregoing studies [20] that diazotrophs can allocate relatively more N to the P-uptake machinery than ordinary phytoplankton under nutrient-depleted conditions. This is of relevance because P-depletion demands greater N investments into nutrient uptake machinery [21,22]. This paradigm is implemented by (1) setting equal grazing preferences for diazotrophs and ordinary phytoplankton conforming with the additional constraint of $\theta_d + \theta_o = 0.4$ and (2) tuning the half-saturation constant for phosphate limitation of diazotrophs k_P^d (where the values considered are smaller than the original value). Again, C_d is varied in addition to minimize the misfit to observations. In total we explore 24 values for k_P^d and C_d. The respective parameter ranges are listed in Tab. S2 and were designed to ensure that diazotrophs have an additional competitive advantage over other phytoplankton in our model. This sets a limit on preferential grazing of 0.2 and 0.044 for the half-saturation constant for PO_4 for diazotrophs. The half saturation constant can not reach zero. Making selective grazing even stronger than in our study leads to an unrealistic die

out of other phytoplankton (e.g. in the upwelling region of the equatorial Pacific). The same holds when increasing C_d above 0.6. In the following OLIGO refers to the most realistic member of the set of 24 that, according to the root mean squared error (RMSE) of simulated nitrogen fixation relative to the considered recent estimate [2].

For the model assessment and the parameter choices we consider climatological annual means in quasi-equilibrium under preindustrial CO_2 emissions and exclude solutions with very low biomass of diazotrophs (below an integrated value of 10 $Tg\,C$). Note, that, while using the RMSE is common [23,24], any choice of a model-data misfit metric inevitably adds a subjective element because the respective choice may well affect the results of the optimisation procedure [25,26]. Please note in this context that in terms of misfit to observed climatologies of phosphate, nitrate and oxygen REF, GRAZ and OLIGO perform comparable to one another when compared to the uninterpolated values of the World Ocean Atlas 2005 [27,28] (Tab. S2). All respective differences in the (volume weighted) RMSEs of the three model versions are smaller than the corresponding average (volume weighted) standard deviations of the observations.

In addition to the two optimized model setups described above, we performed two control simulations: the setup CONTR assumes the same grazing pressure on diazotrophs and other phytoplankton and also uses the same PO_4 half-saturation constants for dizotrophs and ordinary phytoplankton. The respective parameter values are listed in Tab. S1. Note that C_d in CONTR has been set to match GRAZ and OLIGO. The final setup DECAY is identical to CONTR apart from denitrification being set to zero in this model setup.

Table S1: Relevant model parameters in the model version CONTR and the setups GRAZ and OLIGO. Bold values refer to parameters obtained during tuning exercises. Note that θ_o has been calculated with the equation $\theta_d + \theta_o = 0.4$. The last column gives the ranges, in which the parameters were varied to minimize the misfit to the independent nitrogen fixation estimate [2]. The values for GRAZ and OLIGO refer to the respective "best" simulations after the parameters were varied independently.

**Param.**	**Description**	**Units**	**CONTR**	**GRAZ**	**OLIGO**	**Range**
C_d	Growth handicap of diazotrophs	unitless	**0.4**	**0.4**	**0.4**	0.3-0.6
k_P^d	Half-saturation constant for PO_4-limitation for diazotrophs	mmol PO_4/m^3	0.044	0.044	**0.009**	0.002-0.044
θ_d	Grazing preference for diazotrophs	unitless	0.2	**0.14**	**0.2**	0.08-0.2
θ_o	Grazing preference for phytoplankton	unitless	0.2	**0.26**	**0.2**	0.2-0.32
J_O^{max}	Maximum potential growth rate of phytoplankton at $0°C$	day^{-1}	0.6	0.6	0.6	-
J_D^{max}	Maximum potential growth rate of diazotrophs at $0°C$	day^{-1}	$C_d\times$ 0.6	$C_d\times$ 0.6	$C_d\times$ 0.6	-
k_{Fe}	Half-saturation constant for iron (Fe)-limitation	mmol Fe/m^3	0.1	0.1	0.1	-
k_N	Half-saturation constant for NO_3-limitation for phytoplankton	mmol NO_3/m^3	0.7	0.7	0.7	-
k_P	Half-saturation constant for PO_4-limitation for phytoplankton	mmol PO_4/m^3	0.044	0.044	0.044	-
α	Initial slope of the P-I-curve	$(W/m^2)^{-1}d^{-1}$	0.1	0.1	0.1	-
T_b	E-folding temperature of biological rates	$°C$	5.65	5.65	5.65	-

Table S2: Model-data misfits (RMSEs) for the quasi-equilibrated simulations under pre-industrial greenhousgas emissions based on the optimized model versions GRAZ and OLIGO in comparison to the original UVic 2.9 model [4]. The RMSE for fixed nitrogen was calculated relative to a recent estimate of nitrogen fixation of the global oceans under pre-industrial conditions [2] (this value was used to select the optimal model versions for OLIGO and GRAZ). The RMSE values for nutrients refer to surface values while the RMSEs for oxygen refer to all depth levels (volume weighted). The model-data misfits for oxygen and nutrients refer to the uninterpolated values of the World Ocean Atlas 2005 [27,28].

Model	fixed nitrogen $[mmol\ N/m^2/yr]$	RMSE NO_3 $[mmol NO_3/m^3]$	RMSE PO_4 $[mmol PO_4/m^3]$	RMSE O_2 $[mmol O_2/m^3]$
UVic 2.9 [4]	**55.4**	2.6	0.3	9.5
GRAZ	**36**	2.6	0.26	9.8
OLIGO	**35**	2.5	0.29	10.0

Climate States

The two sets of tuning experiments start from the equilibrated state of the original model (named *reference run* in the respective publication [6]) and are integrated for 2000 model years with constant, pre-industrial CO_2 emissions. The experiment evaluations in quasi-equilibrium refer to the average of the last 10 years of these simulations, hindcasting historic conditions. (Note that the model might be sensitive to the spinup length). In addition, we will show results from simulations of GRAZ and OLIGO, covering the period 1800-2150. They start with the historical state and are continued with the CO_2 emission scenario RCP 8.5 [29].

Supplementary Figures

The supplementary Figure S1 shows the time evolution of the diazotrophic biomass in the simulations CONTR and DECAY under constant CO_2 emissions. (CONTR assumes identical grazing pressure and PO_4 half-saturation constants for diazotrophs and other phytoplankton and DECAY additionally assumes zero denitrification). The simulation DECAY shows a clear exponential decay in diazotrophic biomass. Fig. S2 depicts the histograms of observed and simulated and diazotrophic biomass for the different model versions. Fig. S3 shows the simulated surface nutrients for the different model versions. Fig. S4 illustrates the projected pattern of diazotrophs and fixed nitrogen and Fig. S5 refers to the projected biomass of diazotrophs when both paradigms are considered.

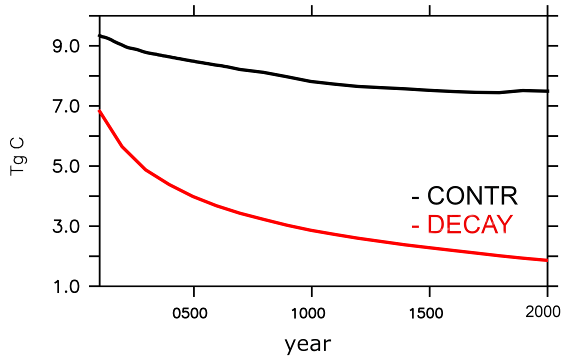

Figure S1: Time evolution of the global diazotrophic biomass (in units TgC) during the spinup in CONTR compared to the simulated biomass in an identical model version where denitrifications was set to zero (DECAY).

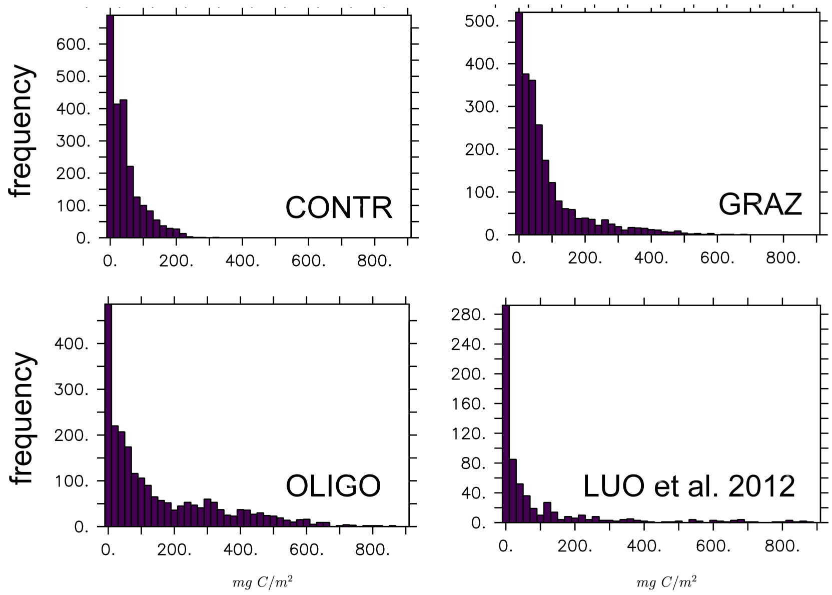

Figure S2: Histograms of the annual mean non-zero diazotrophic biomass integrated over the upper 100m in $mg\,C/m^2$ for the simulations CONTR, GRAZ, OLIGO and the observations [1].

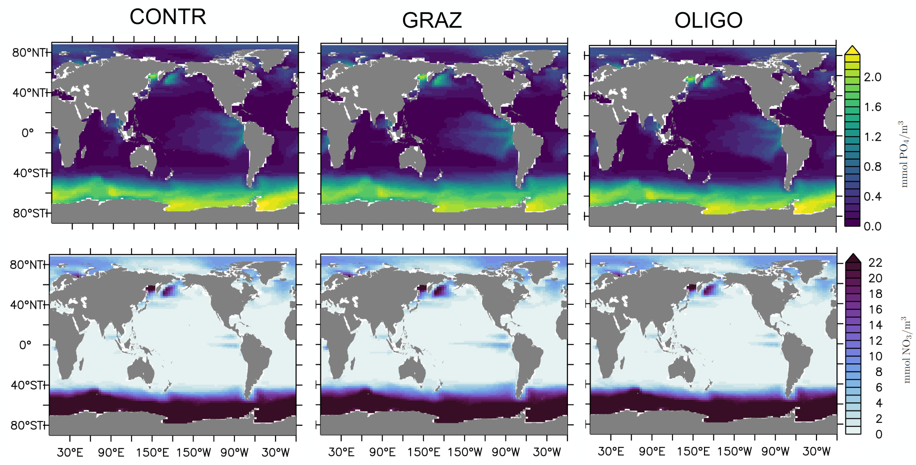

Figure S3: Simulated surface nutrients in the different model versions in historical climate. The upper row refers to phosphate and the lower row to nitrate.

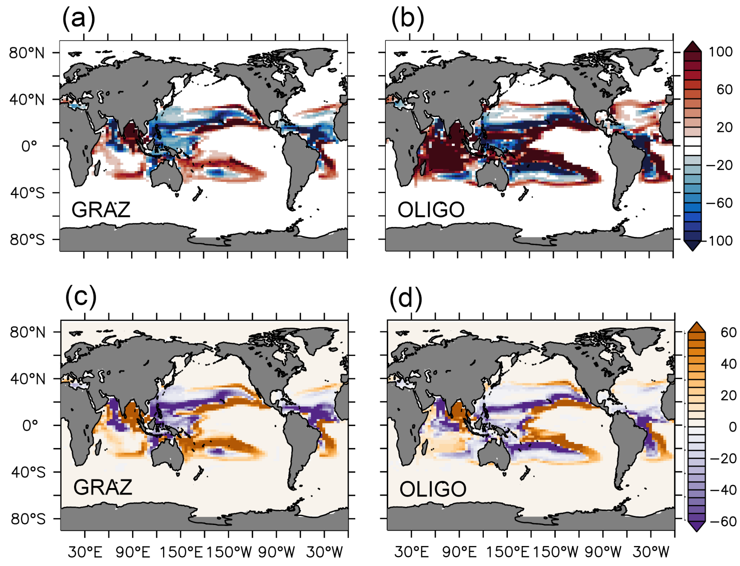

Figure S4: (a) and (b) Projected anomalous annual mean non-zero diazotrophic biomass for the year 2100 relative to 1800 integrated over the upper 100m in $mg\,C/m^2$. (c) and (d) Simulated anomalous nitrogen fixation in $mmol\,N/m^2/yr$ in the year 2100 relative to 1800.

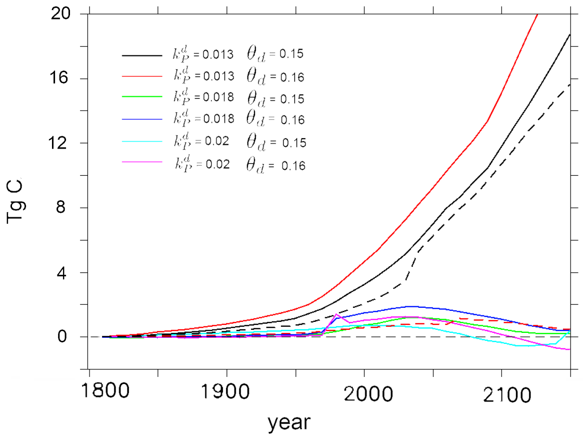

Figure S5: Anomalous projected evolution of (a) global annual mean biomass of diazotrophic biomass in units $Tg\ C$ for various parameter combinations, considering both paradigms. The dashes line show the original simulations GRAZ (red dashed line) and OLIGO (black dashed line).

References

1. Luo, Y. W. *et al.* Database of Diazotrophs in Global Ocean: Abundance, Biomass, and Nitrogen Fixation Rates. *Earth System Science Data* **4(1)**, 47-73 (2012).

2. Wang, W L., Moore, J.K., Martiny, A.C. & Primeau, F.W. Convergent estimates of marine nitrogen fixation. *Nature* **566**, 205-211 (2019).

3. Deutsch C., Sarmiento, J.L., Sigman, D.M., Gruber, N. & Dunne, J.P. Spatial coupling of nitrogen inputs and losses in the ocean, *Nature* **445**, 163-167 (2007).

4. Keller, D.P., Oschlies, A. & Eby, M. A new marine ecosystem model for the University of Victoria Earth System Climate Model. *Geoscientific Model Development* **5**, 1195-1220 (2012).

5. Schmittner, A., Oschlies, A., Matthews, H.D., & Galbraith, E.D. Future changes in climate, ocean circulation, ecosystems, and biogeochemical cycling simulated for a business-as-usual CO_2 emission scenario until year 4000 AD, *Global Biogeochemical Cycles* **22**, GB1013 (2008).

6. Getzlaff, J. & Dietze, H. Effects of increased isopycnal diffusivity mimicking the unresolved equatorial intermediate current system in an earth system climate model. *Geophysical Research Letters* **40(10)**, 2166-2170 (2013).

7. Getzlaff, J. & Oschlies, A. Pilot Study on Potential Impacts of Fisheries-Induced Changes in Zooplankton Mortality on Marine Biogeochemistry. *Global Biogeochemical Cycles* **31(11)**, 1656-1673 (2017).

8. Keller, D.P., Feng, E. Y. & Oschlies, A. Potential climate engineering effectiveness and side effects during a high carbon dioxide-emission scenario. *Nature Communications* **5**, 3304 (2014).

9. Kemena, T.P., Oschlies, A., Wallmann, K.J.G., Landolfi, A. & Dale, A.W. Ocean phosphorus inventory: large uncertainties in future projections on millennial timescales and their consequences for ocean deoxygenation. *Earth System Dynamics* **10(3)**, 539-553 (2019).

10. Mengis, N., Martin, T., Keller, D.P. & Oschlies, A. Assessing climate impacts and risks of ocean albedo modification in the Arctic. *J. Geophysical Research: Oceans* **121(5)**, 3044-3057 (2016).

11. Reith, F., Keller, D.P. & Oschlies, A. Revisiting ocean carbon sequestration by direct injection: A global carbon budget perspective. *Earth System Dynamics* **7**, 797-812 (2016).

12. Landolfi, A., Somes, C.J., Koeve, W., Zamora, L.M. & Oschlies, A. Oceanic nitrogen cycling and N_2O flux perturbations in the Anthropocene. *Global Biogeochemical Cycles* **31(8)**, 1236-1255 (2017).

13. Somes, C.J. & Oschlies, A. On the influence of "non-Redfield" dissolved organic nutrient dynamics on the spatial distribution of N2 fixation and the size of the marine fixed nitrogen inventory. *Global Biogeochemical Cycles* **29(7)**, 973-993 (2015).

14. Weaver, A.J. *et al.* The UVic Earth System Climate Model: Model description, climatology, and applications to past, present and future climates. *Atmosphere-Ocean* **39,** 361-428 (2001).

15. Pacanowski, R.C. MOM 2 documentation, user's guide and reference manual. *GFDL Ocean Group Tech. Rep* **3(3),** 1-232 (1995).

16. Large, W. G., Danabasoglu, G., McWilliams, J. C., Gent, P. R. & Bryan, F. O. Equatorial circulation of a global ocean climate model with anisotropic horizontal viscosity, *J. Physical Oceanography* **31**, 518-536 (2001).

17. Somes, C.J. *et al.* Simulating the global distribution of nitrogen isotopes in the ocean, *Global Biogeochemical Cycles* **24**, GB4019 (2010).

18. Hawser S.P., O'Neil J.M., Roman, M.R. & Codd, G.A. Toxicity of blooms of the cyanobacterium Trichodesmium to zooplankton. *Journal of Applied Phycology* **4**, 79–86 (1992)

19. Kerbrat, A.S., Darius, H.T., Pauillac, S., Chinain, M. & Laurent, D. Detection of ciguatoxin-like and paralysing toxins in Trichodesmium spp. from New Caledonia lagoon. *Marine Pollution Bulletin* **61**, 360-366 (2010).

20. Pahlow, M., Dietze, H. & Oschlies, A. Optimality-based model of phytoplankton growth and diazotrophy. *Marine Ecology Progress Series* **489**, 1-16 (2013).

21. Klausmeier, C.A., Litchman, E., Daufresne, T. & Levin S. A. Optimal nitrogen-to-phosphorus stoichiometry of phytoplankton. *Nature* **429**, 171-174 (2004).

22. Landolfi, A., Koeve, W., Dietze, H., Kähler, P. & Oschlies, A. A new perspective on environmental controls of marine nitrogen fixation. *Geophysical Research Letters* **42**, 4482-4489 (2015)

23. Kriest, I. *et al.* One size fits all? Calibrating an ocean biogeochemistry model for different circulations. *Biogeosciences* **17(12)**, 3057-3082 (2020).

24. Mignot, A., d'Ortenzio, F., Taillandier, V., Cossarini, G. & Salon, S. Quantifying observational errors in Biogeochemical-Argo oxygen, nitrate, and chlorophyll a concentrations. *Geophysical Research Letters* **46(8)**, 4330-4337 (2019).

25. Löptien, U. & Dietze, H. Constraining parameters in state-of-the-art marine pelagic ecosystem models - Is it actually feasible with typical observations of standing stocks? *Ocean Sciences* **11**, 573-590 (2015).

26. Löptien, U. & Dietze, H. Effects of parameter indeterminacy in pelagic biogeochemical modules of Earth System Models on projections into a warming future: The scale of the problem. *Global Biogeocheical Cycles* **31(7)**, 1155-1172 (2017).

27. Garcia, H. E., Locarnini, R. A., Boyer, T. P., Antonov, J. I. & Levitus, S. *World Ocean Database 2005, Volume 4: Nutrients (phosphate, nitrate, silicate.* (ed. Levitus, S.) 1- 396 (NOAA Atlas NESDIS 63, U.S. Government Printing Office, Washington, D.C., 2006).

28. Garcia, H. E., Locarnini, R. A., Boyer, T. P. & Antonov, J. I. *World Ocean Atlas 2005, Volume 3: Dissolved Oxygen, Apparent Oxygen Utilization, and Oxygen Saturation*. (ed. Levitus, S.) 1-396 (NOAA Atlas NESDIS 63, U.S. Government Printing Office, Washington, D.C., 2006).

29. Riahi, K. *et al.* RCP 8.5- A scenario of comparatively high greenhouse gas emissions. *Climatic Change* **109(1-2)**, 33 (2011).
